# Supplementary material for: Value of C-11 methionine PET/CT in patients with intracranial germinoma
Source: PLoS One. 2022 Feb 7;17(2):e0263690. doi: 10.1371/journal.pone.0263690 (PMC8820606; doi:10.1371/journal.pone.0263690)
Supplement: S1 Table — (DOCX) [file pone.0263690.s001.docx]

**S1 Table. Tumor location, SUV_max,_ and T/N ratio of pathologically confirmed eight INGs located in pineal region, sellar-suprasellar region, and basal ganglia.**

| Intracranial non-germinoma | Tumor location | SUV_max_ | T/N ratio |
| --- | --- | --- | --- |
| Two cavernous angiomas | Basal ganglia | 1.66 | 1.53 |
| One glioneuronal tumor | Sellar-suprasellar region | 2.80 | 2.39 |
| One low-grade astrocytic tumor | Sellar-suprasellar region | 2.31 | 1.79 |
| One low-grade glioma | Basal ganglia | 1.94 | 2.16 |
| One immature teratoma | Pineal region | 1.70 | 1.52 |
| One langerhans cell histiocytosis | Sellar-suprasellar region | 3.50 | 3.37 |
| One mixed germ cell tumor | Pineal region | 4.81 | 5.17 |

SUV_max_, maximum standardized uptake value; T/N, tumor-to-normal tissue; ING, intracranial non-germinoma.
