# Supplementary material for: Value of C-11 methionine PET/CT in patients with intracranial germinoma
Source: PLoS One. 2022 Feb 7;17(2):e0263690. doi: 10.1371/journal.pone.0263690 (PMC8820606; doi:10.1371/journal.pone.0263690)
Supplement: S1 Fig — Comparisons of (A) pre-Tx SUVmax and (B) pre-Tx T/N ratio between 22 IGs and eight INGs located in pineal gland, pituitary gland, and basal ganglia during the same study period. (DOCX) [file pone.0263690.s002.docx]

**S1 Fig. Comparisons of (A) pre-Tx SUV_max_ and (B) pre-Tx T/N ratio between 22 IGs and eight INGs located in pineal gland, pituitary gland, and basal ganglia during the same study period.**


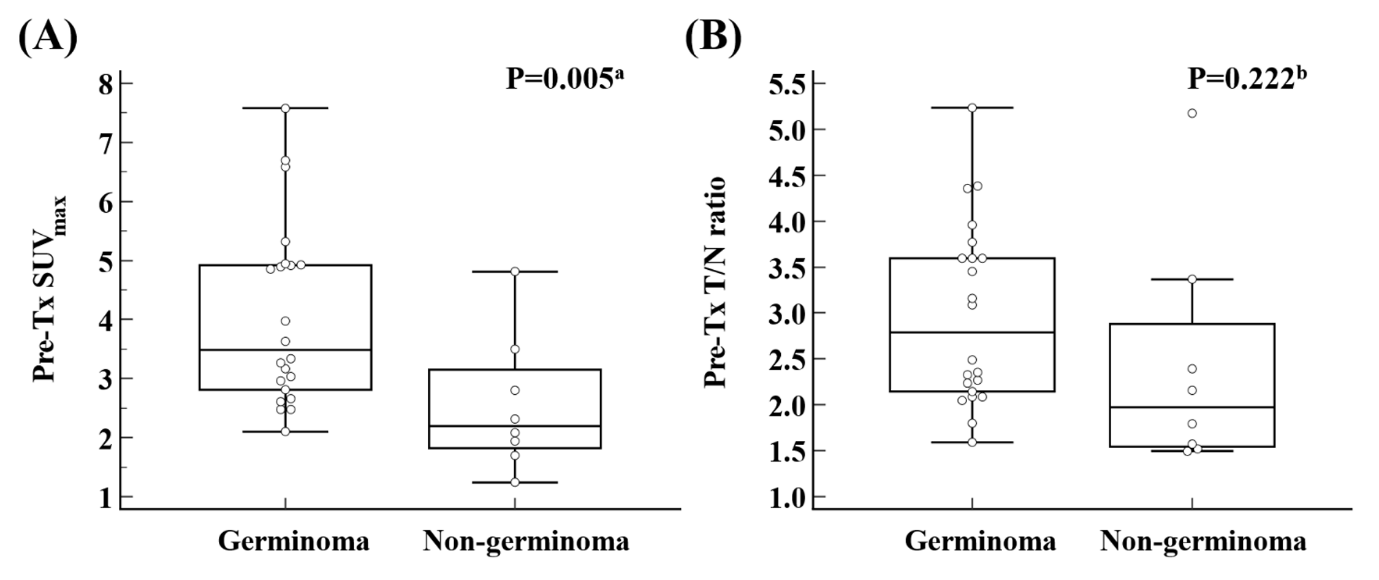


Pre-Tx, pre-treatment; SUV_max_, maximum standardized uptake value; T/N, tumor-to-normal tissue; IG, intracranial germinoma; ING, intracranial non-germinoma.

^a^According to Mann-Whitney U test

^b^According to Independent T-test
