# Supplementary material for: Value of C-11 methionine PET/CT in patients with intracranial germinoma
Source: PLoS One. 2022 Feb 7;17(2):e0263690. doi: 10.1371/journal.pone.0263690 (PMC8820606; doi:10.1371/journal.pone.0263690)
Supplement: Attachment — Submitted filename: Response_to_Reviewers.docx [file pone.0263690.s003.docx]

**Response to Reviewers**

**Reviewer #1:**

This is a very well written paper and deserves only minor changes.

→We would like to thank the reviewer for the careful review of our manuscript and for insightful comments and suggestions, which have helped us improve the quality of the manuscript. We have prepared a point-by-point response to all the comments by the reviewer, and parts that have been corrected or newly added in the manuscript have been written in blue colored font.

The discussion should be implemented with a comparison with FDG.

Reply: Thank you for your valuable comment. According to your suggestion, we added a comparison of FDG and MET PET findings in patients with IGs in the Discussion section.

→ In a previous study of 10 patients with IGs, the IGs could be detected by FDG PET, but tumor-to-background contrast was poor due to FDG uptake by the nearby normal brain tissue [9]. In most of all cases, the FDG uptake of the tumor was intermediate between FDG uptake of the normal white matter and normal gray matter. On the other hand, almost all of IGs could be visualized on MET PET, therefore, MET is considered to be a good radiotracer for detecting IG. In most of all cases, the IGs showed intense MET uptake than the normal white matter and gray matter [9]. Compared to FDG PET, IGs showed relatively low SUV_max_ and relatively high T/N ratios in MET PET [9]. For these reasons, MET PET is considered useful for tumor localization, tumor contouring, and treatment planning (page 14, line 306-313, Discussion section).

The figure legend of fig. 1 should be expanded including also the corresponding T/N ratios and tumor sizes.

Reply: Thank you for your careful comment. As the reviewer suggested, we added the figure legends including tumor sizes, SUV_max_, and T/N ratios.

→ Fig 1. Examples of the three-grade visual scoring system for IG. (A) A case of visual grade 1 was a 24-year-old male patient with pineal germinoma and was post-Tx MET PET/CT images. Tumor size was 9 mm, and post-Tx SUV_max_ and T/N ratio were 1.52 and 1.10, respectively. The MET uptake of tumor was indistinguishable from that of normal brain tissue. (B) A case of visual grade 2 was a 13-year-old male patient with left basal ganglia germinoma and was post-Tx MET PET/CT images. Tumor size was 12 mm, and post-Tx SUV_max_ and T/N ratio were 2.26 and 1.85, respectively. The MET uptake of tumor showed slightly higher MET uptake than that of surrounding normal brain tissue. (C) A case of visual grade 3 was 23-year-old male patient with pineal germinoma and was pre-Tx MET PET/CT images. Tumor size was 19 mm, and pre-Tx SUV_max_ and T/N ratio were 3.97 and 3.45, respectively. The MET uptake of tumor was distinctly higher than that of surrounding normal brain tissue.

IG, intracranial germinoma; post-Tx, post-treatment; MET, C-11 methionine; PET, positron emission tomography; CT, computed tomography; SUV_max_, maximum standardized uptake value; T/N, tumor-to-normal tissue; pre-Tx, pre-treatment; SUV, standardized uptake value (page 6, line 131-142, Figure 1).

Both images should incorporate a color bar.

Reply: Upon your thoughtful comment, we added the SUV color-scale bar (blue/yellow wave, range 0–2.5) to Fig1 and Fig2.

**Reviewer #2:**

The manuscript aims to evaluate the role of [11C]methionine PET/CT in patients with intracranial germinoma (IG). The topic is certainly of interest to readers, as the literature on it is scarce, but the paper needs to be profoundly improved before being considered for publication.

→ We would like to thank the reviewer for the careful review of our manuscript and for insightful comments and suggestions, which have helped us improve the quality of the manuscript. We have prepared a point-by-point response to all the comments by the reviewer, and parts that have been corrected or newly added in the manuscript have been written in blue colored font.

First of all, the language used should be modified in some cases as it does not sound very "scientific" (for example, page 3 line 54 "situation" could be substitute by "clinical scenario" or something similar).

Reply: Thank you for your careful comment. Upon your comment, we revised “situation” to “clinical scenario” in the Introduction section (page 3, ling 54). In addition, we revised some of the manuscript for scientific language.

The introduction section is bare, and many sentences should be further explained (e.g. Why are the tumors occurring at the basal ganglia not easy to distinguish? Or what is the usefulness of MET PET already demonstrated by previous studies?).

Reply: As the reviewer suggested, we added and revised in the Introduction section about why some basal ganglia germinomas were difficult to detect and the usefulness of MET PET reported in previous studies and case reports.

→ MRI is more sensitive than computed tomography (CT) and thus is considered the imaging modality of choice. However, in some cases of basal ganglia germinomas, it was difficult to detect the tumor lesions because the lesions in basal ganglia only show subtle signal change, no mass effects, and nonspecific radiological findings on MRI during their early stages [7, 9-11] (page 3, line 54-58, Introduction section).

→ Previous studies and case reports in patients with IG using MET PET have mainly done research on basal ganglia germinomas [3, 6, 7, 10, 11, 14], and described that MET PET is useful for early detection of basal ganglia germinoma without overt mass formation, assessment of treatment response and residual tumor, and monitoring for tumor recurrence [3, 6, 7, 10]. In previous studies, patients with basal ganglia germinoma presented only with hemiatrophy without any obvious mass formation on MRI, and the studies explained that MET PET was valuable for detecting the precise location of the tumor [10, 11]. In another previous study in IGs, it elucidated the characteristic MET and F-18 fluorodeoxyglucose (FDG) PET findings in 10 patients with IGs and reported that MET is more useful for tumor contouring and treatment planning because MET has better image contrast than FDG [9] (page 3-4, line 64-72, Introduction section).

In the "Materials and methods" section it might be interesting to add the days between biopsy and PET/CT; do you think the uptake could be influenced by a recent biopsy, bearing in mind that the tracer accumulates where inflammatory changes occur?

Reply: We thank the reviewer for bringing this important point to our attention. We also thought that the recent biopsy mentioned in the reviewer's comment can affect MET uptake. In this study, there was a possibility of biopsy-induced inflammation as one of the reasons why MET avidity of basal ganglia germinomas was lower than pineal and sellar-suprasellar germinomas. We summarized these contents and added them to the Discussion section.

→ Furthermore, significant differences in tumor MET avidity were identified according to location. MET PET parameters of basal ganglia germinomas were relatively smaller than those of pineal germinoma and sellar-suprasellar germinomas, while no significant differences were identified between sellar-suprasellar tumors and pineal tumors. These results may be related to whether the patients underwent biopsy prior to pre-Tx MET PET/CT. Of 22 IGs, 17 (77.3%) were biopsy performed prior to pre-Tx MET PET/CT, while the remaining five (22.7%) were pre-Tx MET PET/CT followed by biopsy. Previous studies reported that inflammation may occur due to biopsies in tumors [15, 16], and MET uptake may increase due to the inflammation [17-19]. In all three basal ganglia germinomas in this study, biopsy was not performed prior to pre-Tx MET PET/CT. On the other hand, in 17 (89.5%) of 19 pineal and sellar-suprasellar germinomas, biopsies were performed prior to pre-Tx MET PET/CT. In addition, in this study, mean T/N ratio was high at 3.13 when biopsies were performed prior to pre-Tx MET PET/CT, whereas mean T/N ratio was low at 2.49 when biopsy was not performed prior to pre-Tx MET PET/CT. Therefore, one of the reasons why MET avidity of pineal and sellar-suprasellar germinomas were higher than that of basal ganglia germinomas is possibility of biopsy-induced inflammation. However, in order to confirm this, future MET PET studies in more patients with IG are needed (page 15-16, line 334-348, Discussion section).

Furthermore, what does "MET uptake was markedly higher than background" mean? Did you measure the SUV? If so, how many times did the SUV of the lesion have to be compared to that of BKG to be defined as "markedly higher"?

Reply: Thank you for your valuable comment. In the present study, we performed visual grading analysis of tumors in MET PET/CT through visual assessment. Visual grade 3 of a three-grade visual scoring system defined that MET uptake was markedly higher than background uptake (normal frontal cortex). Through visual assessment, two experienced nuclear medicine physicians classified MET uptake of tumors as visual grade 3 when the MET uptake was higher than background and clearly distinguished from background. When MET uptake of tumors was slightly higher than background and vaguely distinguished from the background, it was classified as visual grade 2. We calculated mean T/N ratios for each visual grade to identify how different each visual grade is quantitatively. In this study, the mean T/N ratio of visual grade 3 was 2.98, and the mean T/N ratios of visual grade 2 and 1 were 1.72 and 1.06, respectively. It means that visual grade 3 was approximately three times higher than background. Therefore, we described "markedly higher" when the T/N ratios of the tumor were about three times the background, and the MET uptake of tumors was clearly distinguished from the background. We added and revised the above description in Material and Methods and Discussion sections.

→ Pre-Tx and post-Tx MET uptake of IGs was evaluated by two experienced nuclear medicine physicians using a three-grade visual scoring system. Grade 1 (negative) indicated that MET uptake was indistinguishable from normal brain tissue, and grade 3 (positive) indicated that MET uptake was markedly higher than background uptake and clearly distinguished from background uptake. Grade 2 (weakly positive) indicated that MET uptake was neither grade 1 (negative) nor grade 3 (positive). Therefore, in the visual grade 2, the MET uptake was slightly higher than background uptake and vaguely distinguished from the background uptake (page 6, line 122-127, Material and Methods section).

→ In all cases in the present study, the tumors showed markedly higher MET uptake than normal white matter and gray matter tissue. Prior to treatment, all tumors were visual grade 3, confirming that MET PET was useful for detection of IGs. This result is in line with a previous study in that the tumors were well delineated on MET PET [9]. We calculated mean T/N ratios for each visual grade to identify how different each visual grade is quantitatively. The mean T/N ratio of visual grade 3 was 2.98, and the mean T/N ratios of visual grade 2 and 1 were 1.72 and 1.06, respectively. This means that visual grade 3 was approximately three times higher than background, and visual grade 2 was approximately lower than twice the background. It was also confirmed that visual grade 1 was similar to background (page 15, line 324-331, Discussion section).

As a final consideration, the sentences regarding study limitations and conclusions are too short and should be improved and the discussion might also include what the future of IG imaging may be, as 11C- needs an on-site cyclotron.

Reply: Upon your careful comment, we added that studies on new PET radiotracers that can overcome the shortcomings of FDG and MET in patients with intracranial germinoma are needed in the Discussion section. In addition, according to your comment, we revised and improved study limitations and conclusion.

→ In previous studies, MET and FDG were mainly used as PET radiotracers in patients with IG. However, in brain imaging, FDG is known to have worse tumor-to-background contrast than MET [9]. In case of MET, the use of MET in medical centers without an on-site cyclotron is restricted because MET has a short radioactivity half-life (20.4 min) [26]. For easy and accurate PET imaging of IGs in more medical centers, future studies are needed for PET radiotracers with better image contrast than FDG and longer radioactivity half-life than MET (page 17, line 390-394, Discussion section).

→ This study has some limitations. First, this was a single-center study with a small number of patients and a retrospective design. Therefore, our results should be verified through future multi-center study involving a large number of patients with a prospective design. Second, in 16 patients (72.2%), biopsies were performed prior to pre-Tx MET PET/CT, and it is possible that the MET parameters of tumor are accompanied by biopsy-induced uptake. Third, MET uptake may be underestimated due to partial volume effect of small lesions less than 2-3 cm in diameter [27]. In the present study, the mean size of IGs was 21 mm (median 18 mm), and several lesions could be affected by partial volume effect (page 17, line 395-401, Discussion section).

→ MET PET/CT has diagnostic value in patients with IGs. In addition, MET avidity is a potential surrogate biomarker of HCG, which has been used as a diagnostic marker for IGs. Tumor MET parameters also had significant differences according to tumor locations, sex, symptoms, and KRAS mutation. However, MET avidity of tumors had no significant prognostic value (page 18, line 404-407, Conclusion section).

**Editor Comments:**

The article is of scientific interest and original to evaluate the role of [11C]methionine PET/CT in patients with intracranial germinoma (IG).

Please follow the reviewer's comments, with particular attention to the semiquantification analysis reporting SUV values and the improvement of the scientific language. Any significant similarity or difference with other similar article should be discussed more extensively as the limitations of the study.

Reply: Thank you for your careful comment. We revised the part that we elucidated about semiquantitative analysis, and revised and improved the manuscript for scientific language. In addition, we elucidated the similarity and difference of this study from previous studies, and we improved study limitations and conclusion.
